# Women and clinicians’ views, preferences and experiences of caesarean section and vaginal birth in India: a qualitative substudy of the ‘Misoprostol or Oxytocin for Labour Induction’ (MOLI) trial

**DOI:** 10.1136/bmjgh-2024-018393

**Published:** 2025-09-05

**Authors:** Kate Lightly, Shuchita Mundle, Jaya Tripathy, Pradeep Deshmukh, Beverly Winikoff, Andrew Weeks, Carol Kingdon

**Affiliations:** 1 Department of Womens and Child Health, University of Liverpool, Liverpool, UK; 2 Obstetrics and Gynaecology, AIIMS Nagpur, Nagpur, Maharashtra, India; 3 Community Medicine, AIIMS Nagpur, Nagpur, Maharashtra, India; 4 Gynuity Health Projects, New York, New York, USA

**Keywords:** Qualitative study, Global Health, Obstetrics, India

## Abstract

**Introduction:**

Caesarean use in India continues to rise and significant disparities exist. However, women and clinicians’ views are under-researched. This paper aims to explore women and clinicians’ views and preferences for mode of birth in two government hospitals in urban central India.

**Methods:**

This qualitative study involved 53 semistructured interviews with high-risk women before and after induction of labour and eight focus groups with clinicians and researchers in two government hospitals in Maharashtra state. All women were recruited to the ‘Misoprostol or Oxytocin for Labour Induction’ (MOLI) randomised controlled trial (NCT03749902) and were induced for hypertensive disorders in pregnancy. Data were analysed using the framework approach to thematic analysis.

**Results:**

Interactions between women, clinicians and families played an important role in women’s birth experiences. Most women strongly preferred vaginal birth. While a vaginal birth was important to women for their long-term health and well-being, the safety of the baby was the priority. Both women and clinicians justified caesarean use to promote fetal safety. Contrary to clinicians’ perceptions, women clearly understood their caesarean indications. The busy clinical environment was an important factor influencing the clinician’s decision and threshold for caesarean. Three themes arose from the data: (1) women’s preference for vaginal birth: a matter of ‘Trouble for two hours or trouble for two months’; (2) clinicians’ perspectives about caesarean use: ‘Don’t take a risk’; and (3) knowledge through experiences and interactions: ‘The pain didn’t come’.

**Conclusion:**

Women strongly preferred ‘normal delivery’ but accepted caesarean birth to promote fetal safety. Clinicians felt labour and vaginal birth were often risky and prioritised fetal safety in this under-resourced context. Women who had a caesarean birth understood their indication for caesarean but, compared with vaginal birth, reported that caesarean caused them additional short- and long-term anxiety, health and social concerns.

**Trial registration number:**

NCT04037683.

**WHAT IS ALREADY KNOWN ON THIS TOPIC**

- Caesarean section rates in India are rising, with significant variations between settings.
- High-quality studies on Indian women’s mode of birth preferences and experiences are lacking.

**WHAT THIS STUDY ADDS**

- Women wanted and valued ‘normal delivery’.
- However, clinicians often felt ‘waiting’ for a vaginal birth was ‘risky’ and used caesarean section to optimise safety where systems and staffing were lacking.

**HOW THIS STUDY MIGHT AFFECT RESEARCH, PRACTICE OR POLICY**

- Renewed vigour and political drive for safe vaginal birth in India is needed to ensure women have positive birth experiences and outcomes.

## Background

 India is one of many countries facing the complex scenario of caesarean section (CS) underuse and overuse.^1^ The national CS rate rose from 2.9% in 1992/1993 to 21.5% in 2019/2021.^2^ In India, the ‘too much too soon, too little too late’ paradigm is evident in huge disparities between government and private hospital CS rates (15% vs 42%) and differences between rural and urban settings (15.2% vs 26.3% in 2016 in Maharashtra).^2^,^4^

In certain circumstances, CS can be life-saving. For ambiguous or non-medical indications, the risk-benefit ratio is less clear. CS is associated with increased maternal risks such as haemorrhage, infection, longer hospital stays and higher out-of-pocket costs,^5^ as well as increased infant-altered immune responses, increased atopy and decreased gut microbiome diversity.^6^ The reasons for the rising CS rate are complex and numerous; they are medical, societal, cultural, political and financial.^7^ Health systems also play an important role as logistical and financial incentives often favour CS, with higher fees for CS and without staffing models to support continuous one-to-one care in labour.^7 8^

Successive global health systematic reviews show most women prefer vaginal birth, and many of these women associate CS with fear and loss of control.^9 10^ It remains a notable gap that while there are multiple qualitative studies on this topic from other low- and middle-income countries (LMICs), such as Iran,^11^ Indonesia^12^ and China,^13^ there are still, to our knowledge, no previous studies from India. Vaginal birth can be seen as ‘natural’ and even a ‘transcendent and empowering’ experience.^9 10^ However, as CS becomes safer and more accepted by women and society, women’s preference for CS is also increasing globally.^14 15^ One meta-analysis found an overall preference for CS in 16% of women, with higher preference rates in middle-income countries (22%).^16^ Other systematic reviews of preferences for mode of birth quote a median rate of CS requests for nulliparous women in the absence of clinical indication as 9%^17^ and 0.3–14% in another.^9^ A thematic synthesis of women’s mode of birth preferences included 52 studies, including 24 from LMICs, and concluded that major factors for some women choosing CS were ‘deep-rooted fears towards vaginal birth’, including pain, injury to the mother or child and loss of control. The uncertainty of vaginal birth and perceived advantages of CS included planning, reduced anxiety and more fetal safety.^10^

Most obstetricians also prefer vaginal birth in the absence of risk factors for CS.^18^ However, the fear of litigation, perception of CS being a ‘safe’ option for childbirth, obstetricians’ convenience and women’s request for CS have all been highlighted in literature reviews.^19^ Themes in a Vietnamese study highlight the ‘mental strain of obstetricians’.^20^ Some women also describe that clinicians prefer CS due to the ‘combination of uncertainty, fear, and medical and non-medical information against vaginal birth’ and their fear of blame.^10^

Despite India being one of the most populous countries on earth and CS the most common operation, there is very little published literature on Indian women’s mode of birth preferences and experiences. Questionnaire surveys have found most women preferred vaginal birth, viewing it as natural and acceptable,^21^ and another study showed that women did not know much about mode of birth.^22^ No qualitative studies from India were included in any of the multinational reviews. India is geographically large and culturally complex, which brings significant health system challenges. Alongside the huge population and disease burden, there are cultural hierarchies related to gender and caste, coupled with staff shortages in the health sector. The role of the midwife in India is negligible, although in recent years there has been some political movement towards developing this role.^23^

As the WHO now focuses on thriving through birth experiences^24^ and the respectful care agenda^25^ is implemented, understanding women’s perspectives and experiences is paramount. This paper aims to explore both high-risk women and clinicians’ perspectives and preferences on mode of birth from two large urban government hospitals in central India. The women in this study all underwent induction of labour (IOL) for hypertensive disorders in pregnancy as part of a randomised controlled trial (RCT) of labour induction methods. They were therefore planning vaginal birth but were also at much higher risk of poor maternal and neonatal outcomes and CS than the background population.

## Methods

### Study design

**Figure 1:**
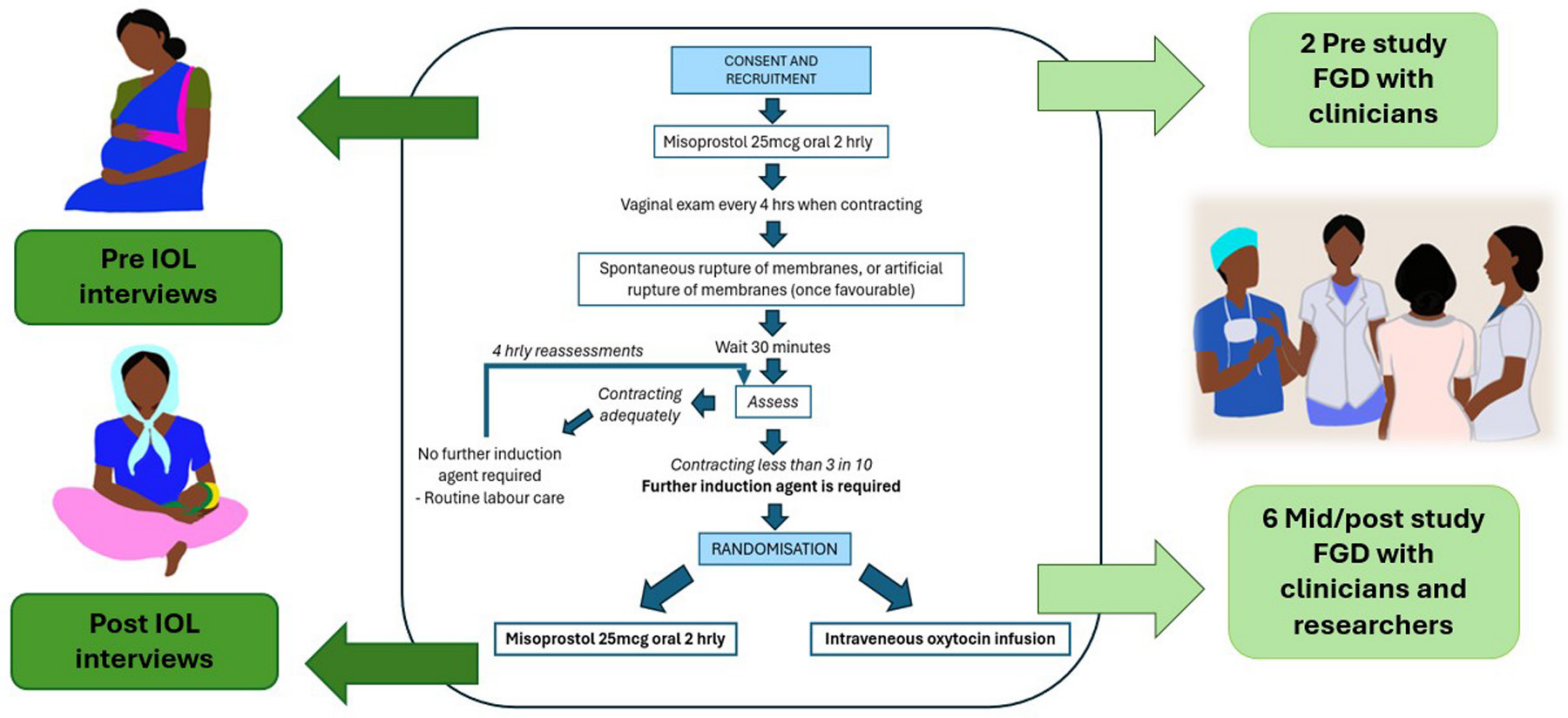
Study diagram. FGD (focus group discussion), IOL (induction of labour)

An alongside qualitative study using semistructured interviews and focus group discussions (FGD) was conducted between October 2019 and December 2021, with two FGDs before the RCT and six during the RCT. The study was paused due to the global COVID-19 pandemic from April to October 2020. Figure 1 shows the study design. This study was part of a larger programme of research. It ran alongside the Misoprostol or Oxytocin for Labour Induction (MOLI) RCT^26^ which compared these induction agents. This study is reported following the Consolidated Criteria for Reporting Qualitative Research guidelines (online supplemental file 1).

### Theoretical framework

The underpinning philosophical approach to this study was aligned with pragmatism. In social theory, pragmatism focuses on the development of knowledge, which it views as social by nature, transcending the dualisms of positivism and ideas of the interpretative and structuralist perspectives.^27^ This study was also pragmatic in its use of theory, based on the mixed epistemological backgrounds of the research group.

### Reflexivity

The research group has mixed backgrounds (obstetricians, community medicine and sociologists) with mixed cultural backgrounds (UK and Indian) and experience in qualitative research. Different team members had different metatheoretical backgrounds and stances, which we aimed to acknowledge through reflexive accounting,^28^ meticulous memo writing and reflection, regular and open meetings and emersion in the culture and project.

### Study setting

This study was conducted in two government hospitals in Nagpur, Maharashtra, central India. The first site was a government tertiary referral hospital and postgraduate training institution, with over 10 000 births annually. The second site was a large government stand-alone maternity hospital, with a lower risk population. Care is doctor led, with no midwives, a high workload and a shortage of human and physical resources. The CS rates are around 40%. Fetal monitoring is primarily intermittent auscultation, with intermittent access to cardiotocography.

### Sampling

Sampling was purposive and conducted according to a sampling frame defined in the study protocol including hospital site, parity, mode of birth, social strata, RCT arm and cadre of staff (ClinicalTrials.gov: NCT040037683). The sampling frame aimed to gather data from a spectrum of women—those with varied experiences and medical and socioeconomic backgrounds—who give birth in urban government settings, immediately before and after birth. This hospital admission is the key time when many interventions relevant to mode of birth occur, the research is feasible and recall bias is reduced. Some women were also interviewed both before and after birth to capture changes in feelings, priorities and experiences for individuals. These women were all high risk, which means a CS birth was more likely, alongside other interventions during birth. The interviews were continued until data saturation was met^29^,^31^ for each aspect of the study. All women were also recruited to the ‘Misoprostol or Oxytocin for Labour Induction’ RCT and therefore also met the ‘MOLI’ inclusion criteria and had a live fetus, with hypertensive disorders of pregnancy, before/after induction of labour and over the age of 18. Women with a previous CS or multiple pregnancies were excluded. Patients who were too unwell or in pain when approached were not interviewed.

FGDs were conducted with practitioners who were involved in screening, recruiting, randomising and consenting participants to MOLI RCT. As it is well documented that obstetric views influence CS rates, FGDs were held with obstetrics and gynaecology doctors (senior and junior) and nurses looking after typical patients in government hospitals within the obstetrics department. A separate FGD was held for the MOLI study research associates who spent a significant time in labour wards with women during labour induction and birth and observing both women and clinicians. Each of these groups (women, healthcare professionals and researchers) provided different insights on the subject to enable comparison and contrast.

### Data collection

The MOLI RCT research associates gave general information and patient information leaflet once they had consented to the MOLI RCT. Then if the patient wanted more information, the qualitative research associate came to give the full information on this qualitative study. Later in the study, women with specific characteristics according to the sampling frame were approached, for example, randomised oxytocin, nulliparous or postnatal. The FGD participants were approached through the usual methods of communication within the department: posters, WhatsApp messages and word of mouth. Interviews were conducted before induction of labour and postnatally between day 1 and day 6 following birth.

Data were digitally recorded on two password-protected recorders following the participants’ informed written consent. Semistructured in-depth interviews were conducted face to face, in the language of the patient’s choice (usually Hindi/Marathi), by two trained female Ayurvedic doctors and research associates (RD and PR). FGDs were facilitated by a senior male medical doctor/qualitative researcher (JT). They were conducted primarily in English, with the use of Hindi when the participants preferred and often as the dialogue evolved. It was deemed unfeasible for the study team to return transcripts to participants because women do not routinely return to the hospitals.

### Data analysis

The framework approach to thematic analysis was used (transcription, familiarisation, coding, development of a working framework, application of the framework, charting data into matrices and analysis).^32 33^ This approach was chosen due to its flexibility, the vast dataset and multidisciplinary research group. Data were transcribed and translated. Detailed line-by-line checking, along with reflective notes and memos, was undertaken. The data were then uploaded into NVivo V.12 software for storage and data management. Three research team members independently coded three transcripts (CK, SM and KL), and KL coded the whole dataset at that time point (two FGDs and 10 interviews), line by line, using inductive open coding. Three separate researchers each devised an initial provisional framework, which was then discussed at length and merged over multiple iterations and several consensus meetings. Codes were categorised and defined. This provisional framework was reviewed and agreed on by the wider qMOLI team and applied to the dataset. When all data had been coded, the final iteration of the framework was agreed on by consensus. The data were charted in a framework matrix in Microsoft Excel, including quotes, summaries and analysis of each code. Matrices were discussed further by the research team and mapped into themes. Rigour and transparency were maintained through team meetings every 1–2 months, as well as frequent smaller meetings.

### Patient and public involvement

A scoping exercise was conducted with women and clinicians during study development. The study protocols and planned publications were reviewed by a consumer representative on the MOLI team.

## Results

A total of 53 semistructured interviews were conducted with 45 women: 19 pre-IOL interviews and 34 postnatal interviews. Eight of these women were interviewed both before and after birth. Of the postnatal interviews, 20 women had a CS, and 14 had a vaginal birth (none had an operative vaginal birth). The most common indications for CS were unsuccessful induction of labour (8/20) and concerns over fetal well-being (8/20). Table 1 reports interview participant demographic characteristics. Eight FGDs were conducted with different cadres of clinicians and research staff across two sites (n=83). The first two were conducted prior to MOLI recruitment and six were conducted during recruitment. Table 2 reports focus group participant demographic characteristics.

**Table 1:** Interview participant characteristics for antenatal and postnatal interviews

Antenatal interviews (n=19)		Postnatal interviews (n=34)	
Characteristics	n	Characteristics	n
Location		Location	
Site 1	7	Site 1	20
Site 2	12	Site 2	14
Age		Age	
18–25	13	18–25	21
26–30	6	26–30	12
>30	0	>30	1
Socioeconomic class		Socioeconomic class	
Low	9	Low	14
Middle	8	Middle	11
High	0	High	1
Unknown	2	Unknown	8
Parity		Parity	
Nulliparous	14	Nulliparous	27
Multiparous	5	Multiparous	7
Gestational age (weeks)		Gestational age (weeks)	
<37	1	<37	4
37+	18	37+	30
		Mode of birth	
		Vaginal	14
		Caesarean	20
		Day postnatal at interview	
		0–1	7
		2–3	17
		4–5	10
		Number of interviews	
		1	26
		2	8

**Table 2:** Focus group participant details

FGD No	Site	Cadre	Participants (n)	Duration (min)	Date
1	Site 1	Doctors—senior and residents	13	49	19 Oct
2	Site 2	Mixed—doctors and nurses	13	44	19 Nov
3	Site 2	Nurses	16	38	21 Sep
4	Site 2	Doctors—senior and residents	10	33	21 Sep
5	Site 1	Doctors—senior	8	36	21 Oct
6	Site 1	Nurses	6	30	21 Oct
7	Site 1	Doctors—residents	7	42	21 Oct
8	Uni	MOLI research associates	10	70	21 Nov

FGD, focus group discussion; MOLI, Misoprostol or Oxytocin for Labour Induction.

**Figure 2:**
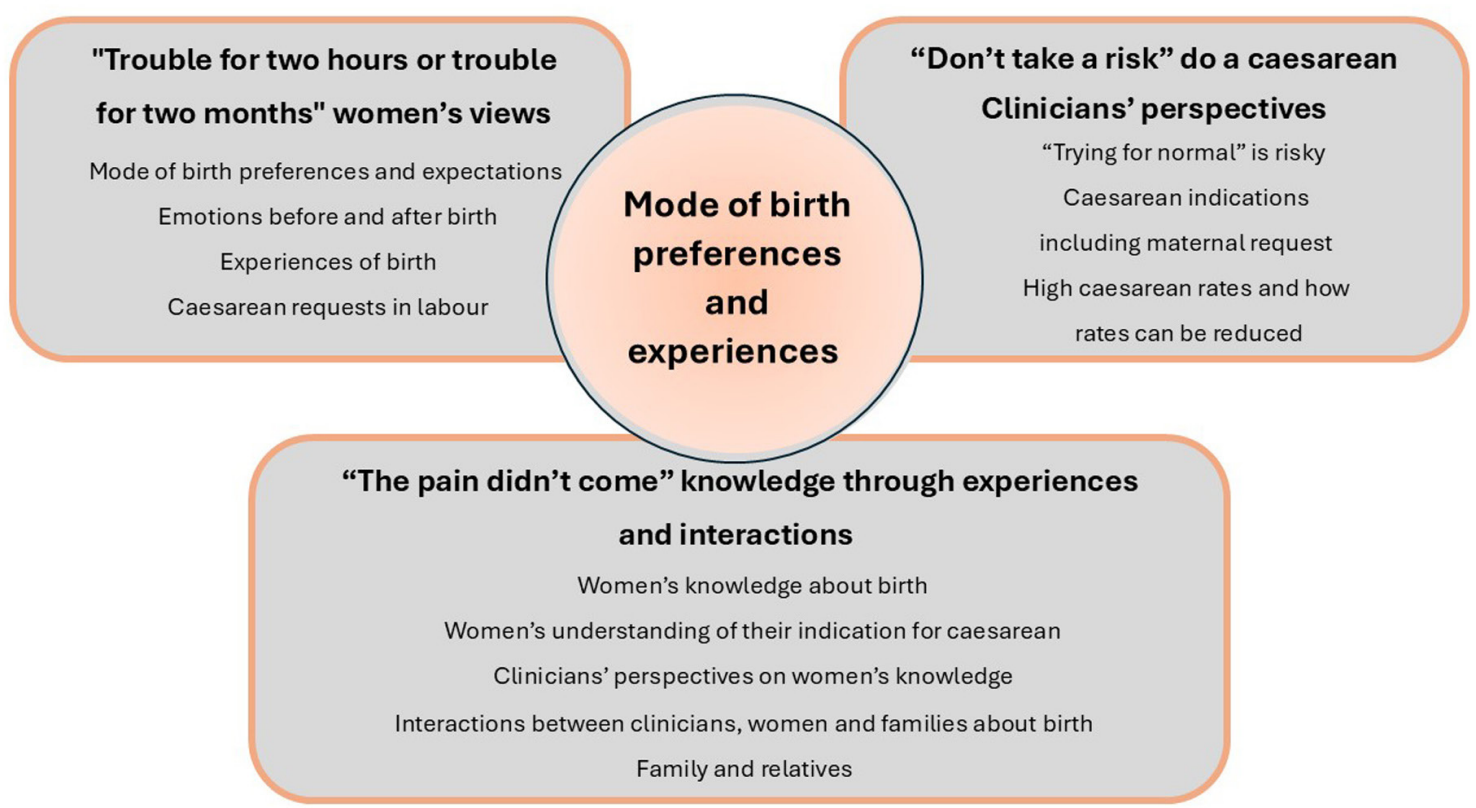
Theme diagram

Three themes and subthemes are reported and shown in figure 2 (overview of themes). Online supplemental file 2 reports the full themes, subthemes and illustrative quotes. Commonly used phrases throughout the dataset are used throughout this paper in quotation marks.

### Theme 1: women’s preference for vaginal birth—a matter of ‘Trouble for two hours or trouble for two months’

The mode of birth and safety of the baby were the most important aspects of birth for women in this study. Most women had a very strong preference for vaginal birth.

#### Mode of birth preferences and expectations

Almost all women strongly preferred ‘normal delivery’ and expected to have a vaginal birth. After vaginal birth, the woman was typically expected to be ‘well’ and healthy, and there was no pain/‘Traas’. Women said they would be able to walk on the first postnatal day, leave the hospital on the third day and slowly undertake their household responsibilities. There were no further worries about issues in the future, and she would be ‘good for her whole life’. Clinicians also knew that women preferred vaginal birth.

For women who underwent CS, there were no reported issues—such as ‘trouble/pain/problems’—at the time of the procedure, and only minimal discomfort or ‘not much trouble’ during the first few days. However, many described experiencing prolonged difficulties afterward, ranging from ‘months’ (descriptions varied between 2 and 10 months) to a ‘lifetime of trouble’. Persistent ‘tension’ was recurring. The ‘troubles’ described include more pain, restrictions on mobility, leaving home and diet. Women often required assistance from others for several months, and some reported difficulties feeding and providing ‘proper attention’ to the baby.

Because everyone says the pain of normal delivery remains till the baby comes, and after Caesar operation, it remains ‘til two months. Means I wanted it for a short time only and didn't want long-term pain. Therefore, I was thinking of normal. (Antenatal woman, P46)

#### Emotions before and after birth

Before birth, women had many anxieties about labour and birth and whether the mother and baby would be well. Many felt scared and afraid, especially when CS was needed. After birth, these worries quickly dissipated. Women who had vaginal deliveries often felt good and were happy to have birthed vaginally and quickly. However, most women reported more negative feelings after CS, primarily due to issues related to the CS.

I would have been very happy if I didn’t have a Caesar. But now I had a Caesar. Everyone was saying that don’t do this, don’t do that, it’s not allowed, that’s not allowed, sit like this, sit like that, don’t eat this, don’t eat that. Means there are lots of things (restrictions)… means feels as if I am stuck in a cage. (Postnatal woman, P37)

#### Experiences of birth

Women typically described their own birth as ‘good’. If the woman and baby were both well, women expressed satisfaction with their birth experience. Similar language and concepts were used by women before and after birth. Pain and trouble (‘Traas’) were more strongly described postnatally, especially labour pains which were very severe and caused significant ‘suffering’ and a ‘one-time’ pain. Many women described ‘fearful’ experiences such as fear before CS and watching others in the labour ward. Women who underwent CS had more negative experiences during labour and postnatally. Despite these negative perceptions, those who had CS had accepted it because it was recommended by the doctor and would also do as the doctor recommended for future deliveries. Some expressed it would have been better if they instead had a vaginal birth, whereas others felt that they should ‘let it be’ and that their mode of birth was out of their hands.

No one regretted vaginal birth. There were no reports of personal verbal abuse or physical violence.

It was good…Now, feeling better. Now, feeling very nice as the baby is delivered. By seeing the baby, we remain happy. Means that whatever worry was there, we forgot all and paid attention to our baby…We keep aside our pain and pay attention to the baby nicely. (Postnatal woman, P24)

#### CS requests in labour

There are some reports of women requesting CS during labour from both women and clinicians; sometimes, the relatives ask doctors, too. However, all the requests from the women interviewed were based on being unable to bear the terrible pain or fear after seeing other women give birth. These were requests for CS as a rescue from the pain or fear rather than a mode of birth preference. Mode of birth preferences were clearly for vaginal birth throughout the data, before and after birth for most women.

At the last, I myself told them that I don’t want [normal] then what they people could do. I insisted, said that I would not bear the pain… like this.

RA—Pain? Would not bear the pain like this.

RA—Okay. Hmm.

So, I myself said to do my caesar. (Postnatal woman, P18)

Women had to ‘bear the pain’ until vaginal birth, then they would have no future concerns related to their birth. For CS, there was ‘a lot of pain’ and trouble ‘traas’, which lasted for months or even longer. Worries for women who had CS did not dissipate after birth due to pain and restrictions.

### Theme 2: clinicians’ perspectives about CS use—‘Don’t take a risk’

For clinicians, while vaginal birth was preferred, ‘waiting/trying for normal’ birth in high-risk women was often considered ‘a risk’.

#### ‘Trying for normal’ is risky

Cultures on risk varied across different institutions; one FGD described that ‘waiting/trying for normal’ birth in women with risk factors such as pre-eclampsia or poor obstetric history was considered ‘a risk’. On the other site, clinicians expressed that vaginal birth was preferred and that ‘we give trial of normal’ (labour/birth). In private settings (where FGD participants also worked), CS was the default option in high-risk women. The research associates involved in the clinical study described a shift in practice for patients with hypertension in one site, away from automatic CS towards IOL and ‘trying for normal’ as the study progressed.

They people [private provider] said that we can’t take the risk of normal. They said that your vein may burst [bleed on the brain]. I was like scared. (Postnatal woman, P12)

#### CS indications including maternal request

CS was only done if there was a ‘clear-cut’ indication and, although some patients asked for CS during labour, maternal request CS was not practised. However, when patients presented with a bad obstetric history (including miscarriages, stillbirth and infertility), a CS was often performed even though these indications for CS are not universally recommended.

As in this hospital, we do not have practice of caesarean by choice. (FGD 5, P3)

#### High CS rates and how CS rates can be reduced

Clinicians perceived that CS rates were high and felt that the CS rates were inevitably increased in government hospitals due to the combination of high-risk referrals, previous CS and free government maternity services. Perceptions were more mixed on the CS rate for low-risk women, with many feeling the CS rate was still too high.

A wide range of issues were raised in the FGDs, ranging from antenatal care and education of women to workload and availability of senior staff and lack of midwives. System issues included appropriate referrals and ‘multiple…technical, logistical and administration’ issues. The excessive workload was described by many as being the main issue. More ‘facilities’ were needed, such as the ability to provide ‘painless birth’ (regional anaesthesia), external cephalic version for breech babies (using abdominal massage to turn babies) and midwives.

The rate is going to be high because of this problem. Because of the referrals that we get, it’s going to be high. (FGD 5, P7)

Despite vaginal birth being well documented as the safest mode of birth universally, in this setting, clinicians often felt that CS offered less risk for many women.

### Theme 3: knowledge through experiences and interactions—’The pain didn’t come’

When women were asked to recount their induction and labour experiences, most women could accurately explain the indication for their CS, for example, failed induction ‘the pain didn’t come’. Paradoxically, when women were asked directly, especially primigravidas, they frequently used the expression ‘I don’t know’. In the pretrial FGDs, clinicians felt that women did not know and could not understand their CS indications. This belief was supported by the women themselves who also believed they were not knowledgeable.

#### Women’s knowledge about birth

Women’s understanding of birth was gained from their own birth experiences and from talking with relatives and friends, witnessing relatives and friends’ birth experiences and watching other women in the labour ward. Direct conversations about birth were often avoided as they were thought to instil fear.

Women might simply say, ‘It was normal’. Women were broadly aware of ‘normal delivery’ and CS, but only a small number mentioned forceps/ventouse. A minority of women had seen vaginal birth on the internet, but many avoided this to avoid getting frightened. Only one participant had gained knowledge through formal education, and information gathering from healthcare professionals on labour and birth was not mentioned throughout. However, this was not a formal question within the interview schedule.

No one asks such things. Till now, whoever asked said only that it’s normal. (Postnatal woman, P45)

#### Women’s understanding of their indication for CS

Although some women did not know the indication for their CS, most could clearly explain it. Indications included ‘meconium-stained liquor’, ‘high BP’, ‘no labour pains’, ‘no labour progress’, ‘transverse lie’, ‘low fetal heart’ and ‘the head is stuck’. These indications broadly matched the indications recorded by the research associates from the patient’s records and demonstrated an understanding of the indication for CS.

‘There is no progress,’ ‘BP is raised,’ ‘baby passed stool in abdomen,’ and ‘pain didn’t come.’ (Postnatal women; 1B, 2, 5, 24)

#### Clinicians’ perspectives on women’s knowledge

Clinicians felt that women did ‘not know’ or ‘understand’ due to the ‘class’ of women who attend government hospitals. ‘Detailed counselling’ was not undertaken. Some clinicians felt that if more time were spent counselling a particular woman, she would be more worried and concerned there was something seriously wrong with her or her baby.

The counselling part is very much neglected in our setup. Usually, the patient is ill-informed because they don’t understand. Mostly the kind of class we get here, they usually don’t understand these things. (FGD 1, P3)

#### Interactions between clinicians, women and families about birth

Women outlined numerous interactions around the mode of birth. Many included ‘giving a [induction of labour] pill’ to ‘try for normal’ birth. However, if ‘BP was raised’ or ‘pain doesn’t come’, a CS would be done. Several women had been told in the private or referring institution that ‘yours will be CS’. ‘Yours will be normal’ was more of a plan for induction leading to vaginal birth than an assurance about the mode of birth. However, some reassurance was also given; ‘all is well, don’t worry’. Conversations about risk, danger, the potential for death and serious complications for mother and baby were also reported in this high-risk group.

Madam said we will try our best to deliver normally. If it’s not done, then we will do your Caeser. But don’t be afraid. (Postnatal woman, P28)

#### Family and relatives

During the birth, relatives had key roles in decision-making, experiences, support, advice and knowledge gain. They typically also prefer ‘normal delivery’, although there were some reports of relatives pleading with doctors to do a CS to ‘end the pain’. Some women discussed birth with relatives who had experience of birth, such as sisters, cousins and some friends. However, many women had not discussed birth with anyone. Women had also seen their relatives’ postnatal struggles first-hand at their homes, especially after CS.

So, my husband was not ready to sign [CS consent]. He was very much scared. He was saying that I’ll not sign. Then, my family pressurised said that you have to sign; otherwise, they will not do delivery. And otherwise, anything will happen, you do or not…They didn’t tell me all because I might have raised BP. (Postnatal woman, P19)

Women knew more than both themselves and clinicians realised. Interactions between women, their relatives and clinicians were integral in their birth experiences.

## Discussion

Both vaginal birth and the safety of the baby were important to participants in this study. Women and clinicians both justified CS to promote fetal safety even though most women did not want a CS. However, many clinicians felt they could not ‘take a risk’ and often opted for a CS in this high-risk population. Most women clearly understood their indication for CS, despite the doctors’ misconception that the women could not understand. Interactions between women and clinicians played a key role in shaping women’s birth experiences. This study adds in-depth qualitative data from women and staff in India about mode of birth. Understanding women’s views and priorities around birth is particularly important in this context, where gender inequity prevails. It provides policymakers with much-needed data to support the respectful care agenda. This study also provides insights into mode of birth, gender roles and expectations of women after birth in the Indian context. Resumption of household responsibilities and newborn care are more difficult following CS birth and have implications for long-term health and future pregnancies.

There are few high-quality qualitative studies available on clinicians or women’s understanding, preferences, priorities and experiences of mode of birth from around the world, and none from India.^10^ Understanding women’s perspectives provides policymakers with much-needed data to inform their decisions. This will support the respectful care agenda in this huge country, where gender inequity is an ongoing issue and women’s voices often remain unheard. The research team had varied backgrounds and a great breadth of research experience, promoting trustworthiness and rigour. A relatively large sample was used, allowing comparison and contrast between women and clinicians’ views, both before and after birth.

This study also had limitations. Having a culturally diverse research team has benefits but can present challenges in cultural understanding and translation issues. Furthermore, as all participants were involved in an RCT about IOL methods, all women were at high risk and had already planned vaginal birth. The local team felt, and the FGD data confirmed, that this reflected the typical hospital population, as maternal request CS was not an option, and most women in government hospitals had risk factors. The presence of the study meant that more women had one-to-one care support, and this would have impacted women’s experiences.^34^ The size and diversity of India mean that the experiences of women and staff in two government maternity units in one city may resonate with others. Our findings may be transferable but are not generalisable.

This study supports the existing global literature that most women and clinicians prefer vaginal birth.^9 10^ It adds to the ‘too much too soon’ literature by highlighting that for high-risk women in this setting, without adequate monitoring, NOT doing CS is considered ‘risky’ by clinicians who fear ‘waiting for normal’. This runs contrary to evidence that maternal deaths in LMICs are 100 times higher than in some high-income countries, and a third of all babies in some regions do not survive CS.^35^ This study adds data from Indian women, close to the time of birth, which was previously lacking, highlighting that those giving birth in government hospitals want a vaginal birth, see this as natural and do not want the long-term implications of CS. In this study, requests for CS were related to experienced or witnessed pain and fear rather than the concerns reported in other studies about future sexual function, perineal damage, control^10^ or the influence of social media.^12^ Pain and lack of access to adequate pain relief as a driver of requests for CS during labour was reported in this study and has previously been reported in other LMIC settings (China). Also, in other studies, fetal safety was the primary concern for both women and clinicians. This study adds significant understanding to concepts of women’s knowledge around mode of birth in this setting. The literature base is weak, but there appear to be widespread misconceptions that women in LMICs ‘don’t know’ and ‘can’t understand’.^22^ This study has found that women can understand their indication for CS and are experts in their own birth experience and further outlines the important interactions between women and clinicians around the time of birth, which have been highlighted by other studies as so pivotal.^12^

The clinicians’ data on mode of birth were in keeping with other studies in that they preferred vaginal birth but justified the risks of CS to promote fetal safety. As in other studies, we found that clinicians, especially in private settings, passed on their fears to women.^36^ So, the paradigm has now shifted. Historically, CS was the ‘risky’ option; now, obstetricians fear ‘waiting for normal’ in settings where the quality of care cannot be guaranteed. Obstetricians must realise their role as powerful influencers and decision-makers, and the importance of their own underlying birth philosophy.^37^

As unnecessary interventions rise globally, further studies are needed to understand this complex phenomenon to design appropriate interventions to improve the outcomes and experiences of women globally. Indian and international policymakers and clinicians must take steps to mitigate the rising CS rates. For many women, CS is not the safest mode of birth. In this setting, it is also not what women want. The dominant narrative is that obstetricians in India primarily do CS for convenience and ease. However, these data demonstrate the realities and perceived risks of providing safe labour care for so many high-risk women at once. In many circumstances, clinicians perceive that CS is safer for the fetus and is one way to ensure a good perinatal outcome. Although neither is easy to achieve, this will only change with a greatly improved quality of maternity care and a reduction in societal and legal blame towards clinicians for bad outcomes.

## Conclusions

Women strongly preferred ‘normal delivery’ but accepted CS to promote fetal safety. Clinicians prioritised fetal safety and felt that ‘waiting for normal’ birth was often ‘risky’. Women understood the indication for their CS, but felt that CS birth caused more ‘traas/trouble’, including additional anxiety, longer term health and social problems—such as caring for their newborn, resuming household responsibilities and pain. For women who underwent a CS, the ‘traas/troubles’ often persisted for months or even years, unlike those who gave birth vaginally, whose difficulties tended to dissipate soon after delivery.

## Supplementary material

10.1136/bmjgh-2024-018393 online supplemental file 1

10.1136/bmjgh-2024-018393 online supplemental file 2

## Data Availability

All data relevant to the study are included in the article or uploaded as supplementary information.
