# Supplementary material for: Women and clinicians’ views, preferences and experiences of caesarean section and vaginal birth in India: a qualitative substudy of the ‘Misoprostol or Oxytocin for Labour Induction’ (MOLI) trial
Source: BMJ Glob Health. 2025 Sep 5;10(9):e018393. doi: 10.1136/bmjgh-2024-018393 (PMC12414161; doi:10.1136/bmjgh-2024-018393)
Supplement: online supplemental file 1 [file bmjgh-10-9-s001.docx]

Supplementary file. COREQ

| Domain 1: Research team and reflexivity | | |  |
| --- | --- | --- | --- |
| *a*). *Personal Characteristics* | | |  |
| 1. Interviewer/ facilitator | Which author/s conducted the interview or focus group? | A local research assistant/ Ayurvedic Doctor named as contributor (RD) conducted interviews with women and author JPT conducted the focus groups with assistance from local research assistants. Another research associate PR conducted the first pre- and post-interview. | Methods |
| 2. Credentials | What were the researcher’s credentials? *E.g*. PhD, MD | The researchers’ credentials are as follows:  KL: MBChB Hons, MRCOG, AFHEA, PhD SM: MBBS, MD  JPT: MBBS, MD  PD: MBBS, MD  BW: BA, MD, MPH  ADW: MB ChB DCH MD FRCOG  CK: PhD  RD: BHMS, MSc  PR: BHMS | - |
| 3. Occupation | What was their occupation at the time of the study? | The researchers’ occupations are as follows: KL: O&G registrar and honorary research fellow  SM: Professor and Head of Department, Obstetrics and Gynaecology  JPT: Assistant Professor, Department of Community Medicine  PD: Professor & Head of Department, Department of Community Medicine  BW: President, Gynuity Health Projects  ADW: Professor of International Maternal Health/Consultant obstetrician  CK: Reader in medical sociology  RD: Ayurvedic Doctor and research associate  PR: Ayurvedic Doctor and research associate | - |
| 4. Gender | Was the researcher male or female? | The researchers’ gender are as follows: KL: Female  SM: Female  JPT: Male  PD: Male  BW: Female  ADW: Male  CK: Female  CK: Female  RD: Female | - |
| 5. Experience and training | What experience or training did the researcher have? | KL was conducting her PhD at the time of the study where qualitative research has been studied in detail with numerous courses and reading. KL lived in India for the set-up and majority of data collection of the study (7 months). SM, JPT, PD and ADW are experienced researchers with many publications and involvement in numerous research methodologies. BW and CK are expert qualitative researchers with numerous high-quality qualitative publications in various settings. | - |
| *b*). *Relationship with participants* | | | |
| 6. Relationship established | Was a relationship established prior to study commencement? | Participants were recruited from the MOLI RCT but there was no prior relationship. | Methods |
| 7. Participant knowledge of the interviewer | What did the participants know about the researcher? *e.g*. personal goals, reasons for doing the research | Participants were aware that interviews and focus groups were for research purposes, alongside the randomised controlled trial on induction of labour methods. They had never met the researcher before. They had information about the study, initially from the MOLI research associates and then further detail from the qMOLI research associates, both verbal and written. | Methods |
| 8. Interviewer characteristics | What characteristics were reported about the interviewer/facilitator? *e.g*. Bias, assumptions, reasons and interests in the research topic | The main interviewer, named contributor (RD), is a local Ayurvedic doctor with considerable experience as a research assistant in many previous studies and communicating with patients sensitively, in her role as an Ayurvedic Doctor.  JT has extensive experience in qualitative research and leading FGDs and was keen to get involved in further qualitative work, as there is limited ongoing in India. | Methods |
| Domain 2: study design | | |  |
| *a*). *Theoretical framework* | | | |
| 9. Methodological orientation and Theory | What methodological orientation was stated to underpin the study? *e.g*. grounded theory, discourse analysis etc | A pragmatic use of theory using the Framework Approach to thematic analysis. | Methods |
| *b*). *Participant selection* | | | |
| 11. Method of approach | How were participants approached? *e.g*. face-to-face, telephone, mail, email | Participants were approached in person once they had consented to the MOLI RCT and provided with a patient information leaflet regarding the purpose of the qualitative study. The MOLI research associates gave general information and then if the patient wanted more information the qMOLI research associate came to give the full information.  Later in the study, women with specific characteristics according to the sampling frame were approached e.g. randomised oxytocin, nulliparous, postnatal.  The FGD participants were approached through the usual methods of communication within the department: posters, WhatsApp messages and word of mouth. | Methods |
| 12. Sample size | How many participants were in the study? | A total of 53 interviews were conducted with 45 women, either before or after induction. 83 doctors, nurses and research assistants were included across 8 focus groups. | Results |
| 13. Non-participation | How many people refused to participate or dropped out? Reasons? | This was a multiple-step approach involving women recruited to the MOLI RCT. Some women did not wish to be included in the qualitative study but there were no women who dropped out once consented. The number of women who declined to participate was not recorded. | Methods |
| *c*). *Setting* | | | |
| 14. Setting of data collection | Where was the data collected? *e.g*. home, clinic, workplace | Data were collected from women within a private room in the clinical area, typically an individual patient room/office. Some interviews were conducted on the ward as per the participant's choice. Focus groups were held in private meeting rooms within the hospitals. The study was based in two government hospitals in a large, central, urban Indian setting (Nagpur, Maharashtra, India); a tertiary referral hospital and a women’s hospital, in a large, central, urban Indian setting, | Methods |
| 15. Presence of non-participants | Was anyone else present besides the participants and researchers? | Most interviews were conducted with only the participant and interviewer (RD) present; however, author KL was present at the first interview and some women wished to have family present – this was noted on the transcript. Focus groups were attended by participants, the interviewer JPT and local research assistants to ensure audio recording was done and consent forms filled in. KL was present for one focus group, and this was noted on the transcript. | Methods |
| 16. Description of sample | What are the important characteristics of the sample? *e.g*. demographic data, date | Women: age, hospital location, socioeconomic class, timing of interview (before or after induction), gestation, parity, induction method, mode of birth, number of interviews  Clinicians: hospital location, role (doctor, nurse, research assistant), timing of focus group (pre-trial or mid-trial) | Tables |
| *d*). *Data collection* | | | |
| 17. Interview guide | Were questions, prompts, guides provided by the authors? Was it pilot tested? | Interview templates were created by authors KL, CK, ADW and SM but reviewed by the full research group. The tool was piloted amongst the research team, and then following the initial interviews, the interview template was reviewed.  Full interview guides were included in the protocol. | Methods  Protocol |
| 18. Repeat interviews | Were repeat interviews carried out? If yes, how many? | 8 women were interviewed both before and after their induction experience. | Tables |
| 19. Audio/visual recording | Did researchers use audio or visual recording to collect the data? | Interviews were audio recorded on two recorders. | Methods |
| 20. Field notes | Were field notes made during and/or after the interview or focus group? | Extensive field notes were made both during and after the interviews and focus groups. | Methods |
| 21. Duration | What was the duration of the interviews or focus group? | The interview length varied between 7 minutes 59 seconds – 41 minutes 49 seconds. Focus group length varied between 30 minutes 24 seconds – 70 minutes 10 seconds. | Tables |
| 22. Data saturation | Was data saturation discussed? | Data saturation was discussed in the methodology section.  Interview templates did involve questions around overall induction experience, mode of birth, fetal monitoring and patient-reported outcome tool MGBSI, so a large number of interviews were needed to meet data saturation. Due to the sheer volume and depth of the dataset, the research team decided to present the various aspects of the work as separate papers. | Methods |
| 23. Transcripts returned | Were transcripts returned to participants for comment and/or correction? | The transcripts were not returned to participants for comment or correction. This was discussed during study planning but deemed unfeasible in this setting, as women do not usually return to hospital.  As a native language speaker, the interviewer did confirm or correct understanding through detailed post-interview discussions which added to our interpretation. | Methods |
| Domain 3: analysis and findings | | |  |
| *a*). *Data analysis* | | | |
| 24. Number of data coders | How many data coders coded the data? | Two researchers KL and CK separately coded the first interviews and devised separate coding frameworks. Through consensus, these coding frameworks were merged. A selection of transcripts (interview 16, 21 and focus group 1) were reviewed by the whole research team, including the interviewer. Emerging codes were added if necessary and presented to the research team regularly. KL then coded the remaining transcripts, with frequent review and edits by CK. | Methods |
| 25. Description of the coding tree | Did authors provide a description of the coding tree? | Yes, primarily inductive approach through open coding of the data. | Suppl table |
| 26. Derivation of themes | Were themes identified in advance or derived from the data | The themes were derived from the data during analysis. These were refined during the drafting of manuscripts across the different publications. | Results |
| 27. Software | What software, if applicable, was used to manage the data? | NVivo 12 | Methods |
| 28. Participant checking | Did participants provide feedback on the findings? | Participant validation was done verbally throughout the focus groups to confirm meaning but formal member checking was not undertaken. Participants did not provide feedback on the findings. However, some women shared their experiences of participating in the study in the interviews, and this was a positive experience for most women. Some clinicians wished to be sent the final publication. | Methods |
| *b*). *Reporting* | | | |
| 29. Quotations presented | Were participant quotations presented to illustrate the themes / findings? Was each quotation identified? *e.g*. participant number | Yes, mostly within Supplementary table with some quotes in the text. Quotes from women were identified by the timing of the interview (prenatal or postnatal) and participant number. Quotes from focus groups were identified by role, focus group number and timing of focus group (pre-trial or mid-trial). | Results  Suppl table |
| 30. Data and findings consistent | Was there consistency between the data presented and the findings? | Yes | Results  Suppl table |
| 31. Clarity of major themes | Were major themes clearly presented in the findings? | Yes, major themes were clearly presented in the findings. | Results  Figures |
| 32. Clarity of minor themes | Is there a description of diverse cases or discussion of minor themes? | Opposing and contradictory views are discussed with illustrative quotes provided where appropriate. Subthemes, or minor themes, are displayed in Table 3. | Results  Suppl table |
