# Supplementary material for: Women and clinicians’ views, preferences and experiences of caesarean section and vaginal birth in India: a qualitative substudy of the ‘Misoprostol or Oxytocin for Labour Induction’ (MOLI) trial
Source: BMJ Glob Health. 2025 Sep 5;10(9):e018393. doi: 10.1136/bmjgh-2024-018393 (PMC12414161; doi:10.1136/bmjgh-2024-018393)
Supplement: online supplemental file 2 [file bmjgh-10-9-s002.docx]

Supplementary material. Themes, sub-themes, and illustrative quotes

| Theme 1: Women’s preference for vaginal birth: a matter of "Trouble for two hours or trouble for two months” | |
| --- | --- |
| Mode of birth preferences and expectations | *“Because everyone says the pain of normal delivery remains till the baby comes, and after Caesar operation, it remains ‘til two months. Means I wanted it for a short time only and didn't want long-term pain. Therefore, I was thinking of normal.”*  *Antenatal woman (P46)*  *“Nothing. It should be normal. That's it. All should go well. Baby should remain well, and me too. That's only my expectation…”*  *Antenatal woman (P29)* |
| Emotions before and after birth | *“Means, how will the delivery happen? This was the fear in my mind. I was very much afraid of how the delivery will happen. What will happen?”*  *Postnatal woman (P24)* |
| Experiences of birth | *“It was good…Now, feeling better. Now, feeling very nice as the baby is delivered. By seeing the baby, we remain happy. Means that whatever worry was there, we forgot all and paid attention to our baby…We keep aside our pain and pay attention to the baby nicely.”*  *Postnatal woman (P24)* |
| Caesarean requests in labour | *“At the last, I myself told them that I don't want [normal] then what they people could do. I insisted, said that I would not bear the pain.. like this.*  *RA- Pain? Would not bear the pain like this. RA- Okay. Hmm.*  *So, I myself said to do my caesar. RA- Okay. You said yourself?”*  *Postnatal woman (P18)* |

| Theme 2: Clinicians’ perspectives about caesarean use: “Don’t take a risk” | |
| --- | --- |
| “Trying for normal” is risky | *“They people said that we can't take risk of normal. Said that your vein may burst. I was like scared. “*  *Postnatal woman (P12)* |
| CS indications including maternal request | *“As in this hospital, we do not have practice of caesarean by choice”*  *FGD 5, P3*  *“P3 - Previous scar.*  *P 10 – Sir, most of the patients, or 80% are referrals here. They come in with emergencies*  *P5 – Prolonged labour*  *P10 – Prolonged ROM.”*  *FGD 3, P3,5,10* |
| High CS rates and how CS rates can be reduced | *“M - What's the general caesarean rates in the hospital? Do you think it's (interrupted)…?”*  *P8 - It is very high, 40 to 60%.*  *P7 - Maybe 30 to 40%, and the reason behind this is that this is the referral institute. All high-risk patients are referred here, and they come here for caesarean section because the indication is like that. So, most of the time, cases preeclampsia, eclampsia patients, LSCS, previous LSCS, placenta praevia. These patients are only referred here. So, the indication itself is a caesarean section for these patients.*  *P8 - But, in low-risk patients also, we have a high rate of caesarean section. And I personally think that we don't have, compared to the rest of Nagpur, rest of Maharashtra, normal delivery is quite good. We are not that bad comparatively.*  *P7 - Even with this, the rate is going to be high because of this problem. Because of the referrals that we get, it’s going to be high.”*  *FGD 5, P7,8,9*  *“P3 - It's multiple issues. Multi-problem thing.*  *P8 - The main thing is that the workload. I think everything can be explained on the basis of workload which we have over here. Otherwise, the things are better than any other institute, I think.*  *P2 - And missing those midwives. We don't have midwives. We are all doctors here. They are doing caesarean, everything, right from preparation of the patients everything. Everything is done by the doctors. Many times, nurses are not there. Once the baby is out, they vanish. Right from assisting episiotomy, they don't know anything. That problem is also there…*  *M - Anything that can be done to reduce the rates of caesarean section?*  *P7 - The most important is fetal monitoring.*  *P8 - Better monitoring.*  *P7 - If you can monitor the fetal heart sounds, sincerely and [interrupted]*  *P8 - Better understanding and better training of the like students and the staff will help in reducing the caesarean sections.”*  *FGD 5, P2,3,7,8* |

| Theme 3: Knowledge through experiences and interactions: “The pain didn’t come” | |
| --- | --- |
| Women's knowledge about birth | *“No. Don't have any knowledge about it. No one told us any details. Only told that I had a normal delivery. Told that only (all that is said is I had NVD). And it's said that if pain increases, the baby will soon deliver because of the pain. Then, we have to apply the force (push), so it happens early. Therefore, I liked that.*  *No one asks such things. Till now, whoever asked said only that it's normal.” (Only share it was NVD, no details shared.)*  *Postnatal woman (P45)* |
| Women’s understanding of their indication for CS | *"There is no progress," "BP is raised," "baby passed stool in abdomen", and "pain didn't come."*  *Postnatal women (P1B, 2, 5, 24)* |
| Clinicians' perspectives on women's knowledge | *“The counselling part is very much neglected in our setup. Usually, the patient is ill-informed because they don’t understand. Mostly the kind of class we get here, they usually don’t understand these things.”*  *FGD 1, P3* |
| Interactions between clinicians, women, and families about birth | *“Their importance is that the baby should come out safely, do whatever you want. They are also willing to endure the pain. But baby should deliver well. First, they take guarantee of it.”*  *FGD 4, P8*  *“Madam said we will try our best to deliver normally. If it’s not done, then we will do your Caeser. But don’t be afraid”*  *Post natal woman (P28)* |
| Family and relatives | *“So, my husband was not ready to sign [CS consent]. He was very much scared. He was saying that I'll not sign. Then, my family pressurised said that you have to sign; otherwise, they will not do delivery. And otherwise, anything will happen, you do or not. Then, he signed. So, all prayed in the way of GOD IS ONE (parmatma ek). They didn't tell me all because I might have raised BP.”*  *Postnatal woman (P19)* |
